# Inflammatory Response in Islet Transplantation

**DOI:** 10.1155/2014/451035

**Published:** 2014-04-30

**Authors:** Mazhar A. Kanak, Morihito Takita, Faisal Kunnathodi, Michael C. Lawrence, Marlon F. Levy, Bashoo Naziruddin

**Affiliations:** ^1^Institute for Biomedical Studies, Baylor University, Waco, TX 76712, USA; ^2^Islet Cell Laboratory, Baylor Research Institute, Dallas, TX 75204, USA; ^3^Baylor Annette C. and Harold C. Simmons Transplant Institute, 3410 Worth Street, Dallas, TX 75246, USA

## Abstract

Islet cell transplantation is a promising beta cell replacement therapy for patients with brittle type 1 diabetes as well as refractory chronic pancreatitis. Despite the vast advancements made in this field, challenges still remain in achieving high frequency and long-term successful transplant outcomes. Here we review recent advances in understanding the role of inflammation in islet transplantation and development of strategies to prevent damage to islets from inflammation. The inflammatory response associated with islets has been recognized as the primary cause of early damage to islets and graft loss after transplantation. Details on cell signaling pathways in islets triggered by cytokines and harmful inflammatory events during pancreas procurement, pancreas preservation, islet isolation, and islet infusion are presented. Robust control of pre- and peritransplant islet inflammation could improve posttransplant islet survival and in turn enhance the benefits of islet cell transplantation for patients who are insulin dependent. We discuss several potent anti-inflammatory strategies that show promise for improving islet engraftment. Further understanding of molecular mechanisms involved in the inflammatory response will provide the basis for developing potent therapeutic strategies for enhancing the quality and success of islet transplantation.

## 1. Background

Transplantation of pancreatic islets is a minimally invasive procedure involving infusion of islet cells into the portal vein of the liver, and it was first demonstrated in an experimental diabetic model by Kemp et al. in 1973 [1]. Results from the initial clinical trials for type 1 diabetes were only partially successful, with only 10% of the patients achieving insulin independence at 1 year after transplantation [2]. A quantum leap in islet transplantation occurred when the Edmonton group introduced a steroid-free immunosuppression and showed insulin independence in all 7 of their patients [3]. With this advancement in immunosuppression and the continuous improvement in islet isolation techniques, islet transplantation has entered into a new era of heightened success. Currently, two types of clinical islet transplantation are performed: allogenic and autologous islet transplantation. Allogenic islet transplantation is typically performed on patients with severe type 1 diabetes, while autologous islet transplantation is performed on patients suffering from severe chronic pancreatitis (CP) and undergoing partial/total pancreatectomy.

CP is a progressive inflammatory disease that leads to irreversible damage of the pancreatic parenchyma [4]. In its early stages, the disease affects pancreatic exocrine function, which could be followed by impaired endocrine function that results in the onset of diabetes mellitus [5, 6]. The mechanisms underlying the development of CP are not clearly defined. Inflammation, heavy alcohol consumption, pancreatic ductal obstructions, calcification, sphincter of Oddi dysfunction, certain genetic mutations, and periampullary tumors are considered the major causes of CP. In addition to gradual loss of exocrine and endocrine functions, complications of CP include biliary or duodenal stenosis and intractable pain. Total or partial pancreatectomy followed by intrahepatic transplantation of autologous islets has emerged as a promising approach to treat CP, due to its ability to reduce or eliminate pain while retaining endocrine function. The first pancreatectomy followed by autologous islet transplantation was performed at the University of Minnesota in 1977 [7]. Since then, more than 500 CP patients have successfully undergone this procedure at several centers in North America and worldwide [8]. Posttransplant results have shown that at 3 years, 30% of patients were insulin independent, 33% had partial islet function, and 94% showed improved pain control [9].

Comparison of patients receiving similar doses of autologous versus allogeneic islets has shown that the survival of autologous islets is far better over time [9]. In contrast to allogeneic islet transplants, autologous islet transplants are devoid of autoimmune response and alloimmune rejection and recipients do not require beta-cell-toxic immunosuppressive drugs. Similar to intrahepatic allogeneic islet transplants, however, autologous islet transplants are subjected to ischemic, hypoxic, and innate inflammatory damage.

**Figure 1:**
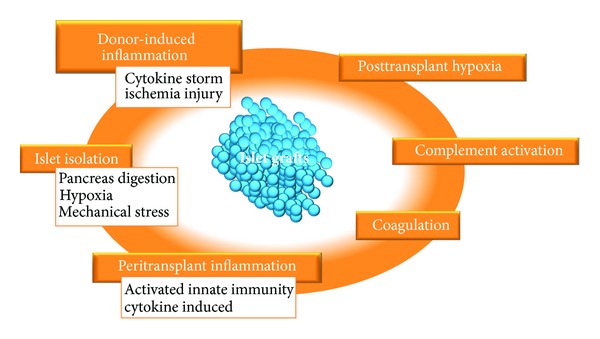
Factors that induce an inflammatory reaction to islet grafts.

Despite recent progress in clinical outcomes, several obstacles for allogenic and autologous islet transplantation remain to be addressed. These include improvement in the technical aspects of the isolation procedure, improvement in the quality of isolated islets, inflammation during the peritransplant period triggered by incompatibility between islets and the blood interface, known as instant blood-mediated inflammatory reaction (IBMIR), and long-term survival of transplanted islets. The common underlying cause that largely contributes to each of the obstacles to clinical success is the production and secretion of inflammatory mediators, which induce apoptosis and impair function of transplanted islets (Figure 1). In this paper, we discuss the causes of inflammation in islets, including inflammation related to donor factors, islet isolation, pretransplant islet culture, the transplant itself, and posttransplant factors.

## 2. Pancreas Donors

Damage to the islet cells begins with the donor. Immune cell infiltrates and inflammatory mediators are produced in the donor pancreas upon brain death. Most islet transplants use organs from heart-beating, brain-dead (BD) cadaveric donors. Organs from BD donors have much better outcomes than organs from non-heart-beating donors. However, acute physiological changes after brain death in BD donors may still result in significant damage to the islets from inflammatory events. Indeed, several reports have documented poor transplantation outcomes due to organ damage caused by extremely high levels of inflammatory cytokines in the circulation [10–13]. This systemic elevation of inflammatory cytokines, the so-called “cytokine storm” in BD donors, is due in part to hemodynamic and metabolic changes occurring after brain death [14]. Inflammatory cytokines are effective mediators of beta-cell dysfunction and death [15].

Recent studies have demonstrated methods to improve islet graft function by inhibiting BD inflammatory mediators upon procurement. One study showed that maintaining normoglycemia after brain death by insulin reduces inflammatory shock [16]. Transfusing sivelestat sodium, a selective neutrophil elastase inhibitor, was shown to increase islet yield, protect islet function and quality, and inhibit hypercytokinemia-mediated beta-cell death in a brain-dead rat model [17].

## 3. Ischemic Time

Islets are subjected to hypoxic conditions from the onset of ischemia up until graft revascularization. Hence, ischemic time is a major concern to be addressed to improve islet quality for transplantation. Since islets are physiologically adapted to receiving an ample supply of oxygen, prolonged ischemic times increase damage to islets. Previous study had shown that isolations performed with pancreas with cold ischemic time of >8 hours produces significantly lower islet yields with impaired islet function [18]. The quality of the islets can be improved by reducing organ ischemic time. Another strategy has been used to reduce islet ischemic damage by maintaining the procured pancreas in a high oxygen content solution. A mixture of University of Wisconsin solution along with perfluorocarbon, which has high affinity for oxygen, has been used to reduce islet damage by hypoxia [19].

The viability of islets is significantly reduced due to depletion of ATP and apoptosis induced by hypoxia [20]. Hypoxia in islets activates NF-*κ*B signaling and induces transcription of inducible nitric oxide synthase (iNOS) and increases expression of MCP-1, leading to infiltration of macrophages and destruction of islets [21]. During hypoxia, islets try to compensate for the low oxygen availability by activating the transcription factor hypoxia-induced factor (HIF) [22]. When activated, the HIF-*α* and HIF-*β* subunits translocate to the nucleus and bind to the hypoxia response element. This induces transcription of several genes, including toll-like receptors. Under normal conditions, HIF activity is rendered inactive by prolyl hydroxylases (PHD) and factor inhibiting HIF (FIH). Both PHD and FIH prevent the activation of I*κ*B kinase B and hence inhibit NF-*κ*B activation [23–27].

## 4. Islet Isolation Process

Isolation of high-quality islets in sufficient quantity remains a major impediment for the further success of this procedure, due to chronic inflammatory conditions affecting the pancreas. The enzymes used for islet isolation could be a factor in the lack of satisfactory results in islet transplantation. It is well documented that enzymatic and mechanical stress can induce inflammatory mediators in islets. Gene array studies have shown upregulation of genes related to inflammation, apoptosis, cell growth, and angiogenesis immediately after isolation, with further increase after 72-hour culture [28]. The most highly upregulated genes have been the ones for inflammation and apoptosis. Isolation and culture of islets have also been shown to downregulate expression of IL-10, which is a negative regulator of cytokine expression and also induces development of regulatory T cells. Conversely, isolation and culture of islets have upregulated the expression of IL-8, which is a potent inflammatory and angiogenic factor, and the expression increased several folds after 3 days in culture [28]. IL-8 has been shown to play a negative role in the longevity of the graft in kidney, liver, and lung transplants; overexpression of IL-8 has been associated with poor graft life [29–31]. Gene array analysis of cultured islets has also shown that the top 15 upregulated genes were all induced by the NF-*κ*B pathway [28]. Therefore, inhibiting the NF-*κ*B pathway during isolation and/or culture might improve islet quality for transplantation.

## 5. Pretransplant Islet Culture 

There has been a continuous debate as to which islets are superior: freshly isolated or cultured islets. Several reports have shown that freshly isolated islets are better than islets cultured for at least 24 hours [32–34]. A recent report from our center confirmed that freshly isolated islets outperform in transplantation when compared to cultured islets to address concerns over the rapid deterioration of islet counts after culture. The study showed a decrease in the islet counts by 13%, 24%, and 35% on days 1, 2, and 3, respectively. When transplanted into diabetic nude mice, the achievement of normoglycemia was significantly improved with fresh islets compared to cultured islets [35, 36]. It has been reported that culturing islets can lead to overexpression of inflammatory mediators caused directly by islets, by stimuli in the culture medium, or by contamination of exocrine tissue in the culture [37]. During culture, islets have to depend on oxygen through diffusion, but the core of the islet begins to undergo hypoxia, causing upregulation of genes induced by oxidative stress and apoptosis [38]. Islets in the culture have been shown to induce expression of proteins such as tissue factor and monocyte chemoattractant protein-1 (MCP-1); this induction could be abrogated by the use of nicotinamide in the culture media [39]. Still, there remains the argument that culturing islets before transplantation will improve the purity of the islets, as acinar cells and damaged islets would die off in the culture. It has also been suggested that providing a culturing period would allow islets to recover from the stress of the isolation process, although there is no compelling data to support this hypothesis. Nonetheless, the Consortium of Islet Transplantation (CIT) has adopted pretransplant culture as a standard procedure for clinical transplants [40].

## 6. Peritransplant Inflammation 

**Figure 2:**
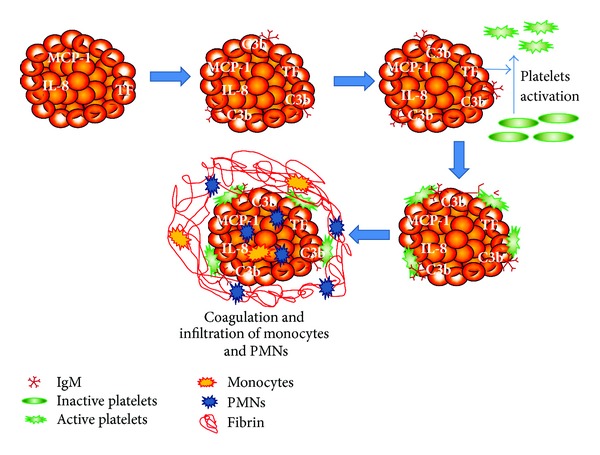
Mechanisms of the instant blood-mediated inflammatory reaction. PMNs: polymorphonuclear cells.

One of the major causes of islet destruction during transplantation is the instant blood-mediated inflammatory reaction (IBMIR) (Figure 2). Bennet et al. first proposed that when freshly isolated “naked” islets come into direct contact with the ABO compatible allogenic blood, activation of innate immune reaction ensues [41]. Tissue factor (TF) expressed on islets could be a major trigger for such reaction. The islets along with resident antigen-presenting cells (APCs) secrete cytokines and chemokines, which play major role in the inflammatory process. Infiltration of leukocytes and macrophages initiate destruction of the islet cells before engraftment [41, 42]. Blocking TF using antibodies significantly reduced the clotting reaction [42]. IBMIR is also characterized by coagulation, complement activation, secretion of chemokines that attract innate immune cells, and release of proinflammatory cytokines leading to islet damage by apoptosis [43].

Although previous studies have shown induction of IBMIR under allogenic and xenogeneic conditions [41, 42], the existence of IBMIR following autologous islet infusion had not been demonstrated in either experimental or clinical settings. Our group recently demonstrated a role of IBMIR in autologous settings [44]. IBMIR was observed in patients undergoing total pancreatectomy followed by autologous islet transplantation. During the initial 0 to 3 hours after infusion of islets, there was a significant increase in markers for coagulation such as thrombin-antithrombin and in proinflammatory cytokines such as interleukin (IL)-6, IL-8, and IP-10 in conjunction with C-peptide, indicating damage to the transplanted islets by the inflammatory response. Further analysis using a miniaturized* in vitro* tube model corroborated the* in vivo* observations and also showed expression of tissue factor (TF) on islets mixed with autologous blood [44]. Therefore, it was concluded that IBMIR is a problem not only in allogenic and xenogeneic islet transplantation but also in autologous islet transplantation.

The IBMIR effect has been characterized by an immediate increase in the thrombin-antithrombin levels after islet infusion and in the levels of C-peptide in the blood. A recent clinical study utilized labeled islets combined with positron emission tomography and computed tomography, and it has been demonstrated that about 25% of the islets are lost immediately after transplantation [45]. The mechanisms underlying IBMIR and methodologies to block this phenomenon are currently being investigated as described below.

### 6.1. Coagulation

Coagulation is activated immediately when the islets come in direct contact with the portal blood during transplantation. The coagulation occurs through the extrinsic pathway, which requires TF for activation. TF expressed on the surface of isolated islets interacts with factor VIIa and initiates the coagulation cascade by formation of thrombin and generation of fibrin clots [42, 46, 47]. The production of thrombin at high concentrations can exacerbate the destruction process because at high concentrations thrombin is proinflammatory and an inducer of apoptosis [48, 49]. Özmen et al. showed that the use of anticoagulants such as melagatran could significantly reduce IBMIR* in vitro* in a blood loop model [50]. Johansson et al. previously showed that low-molecular-weight dextran sulfate (LMWDS), a potent inhibitor of coagulation and complement activation, could efficiently suppress IBMIR. They used an* in vitro* tubing loop model to investigate the effect of LMWDS on islets when exposed to human blood, showing effects on activated partial thromboplastin time, platelet function, and complement activation [51]. Other strategies to block IBMIR that have been intensely investigated have included encapsulating islets or coating the islet surface with inhibitory molecules to promote successful islet engraftment. The islet surface has been coated with heparin complex by exploiting biotin-avidin chemistry to inhibit coagulation [52]. Endothelial cells have also been used to coat the islets; transplantation of these coated islets has reduced coagulation, complement activation, and leukocyte infiltration [53, 54]. Alternatively, the use of mesenchymal stem cell-coated islets has been shown to promote endothelial cell proliferation, revascularization, islet neogenesis, and immune modulation [55]. Another study showed that IBMIR could be targeted by overexpressing CD39, which is an ectonucleotidase that degrades ATP required for platelet activation in the islets.* In vivo* and* in vitro* experiments showed less coagulation activity after transplantation using islets from CD39 overexpressing transgenic mice [56].

### 6.2. Complement Activation

Another major component of IBMIR is the activation of complement cascade. Complement activation is shown by the increase in the concentration of complement proteins in the serum of islet transplant recipients [43, 57]. Activation of complement proteins C3a and C5a leads to recruitment and accumulation of leukocytes, upregulation of adhesion molecules on the endothelium and platelets, and production of reactive oxygen species (ROS) and cytokines [57]. Also, the accumulation of IgG, IgM, and complement proteins C3, C4, and C9 on the surface of human islets immediately after treatment with ABO-compatible blood indicates that immunoglobulin is involved in the activation of the classical complement pathway [57]. Complement protein C5a has been proposed as a critical molecule responsible for activation of coagulation and inflammation by mediating TF expression in neutrophils [58, 59]. A recent short report from Tokodai et al. showed the combined effect of anticoagulant gabexate mesilate and complement protein C5a inhibitory peptide to successfully improve islet transplantation compared to individual use of the drugs in a syngeneic rat transplant model [60]. Thrombin-antithrombin levels were also reduced with the combined treatment. TF was downregulated by complement protein C5a inhibitory peptide treatment, but only on the liver granulocytes and not on the islet grafts [61]. The use of a complement inhibitor such as compstatin has been shown to significantly improve graft survival in* in vivo* models [62]. In a recent study, it has been reported that immobilization of soluble complement receptor on the islet surface using polyethylene glycol inhibited complement activation significantly and protected islets from damage due to xenoreactive antibodies [63]. In clinical autologous islet transplantation, there is no influence of complement proteins during IBMIR [44].

### 6.3. Cytokine Secretion, Leukocyte Infiltration, and Beta-Cell Damage

Syngeneic transplant models have shown that the damage to islet grafts is primarily due to a nonspecific inflammatory response [64, 65]. Similarly, clinical data on autologous islet transplantation on patients with CP showed elevated levels of proinflammatory cytokines immediately after transplantation [44]. The islet resident macrophages, Kuppfer cells, and neutrophils secrete IL-1*β*, which impairs insulin secretion and induces islet cell apoptosis [66–68]. IL-1*β* activates NF-*κ*B signaling and induces expression of several other inflammatory mediators, including IL-1**β**, IL-8, IP-10, IL-6, TNF-**α**, MCP-1, intercellular adhesion molecule-1, vascular cell adhesion molecule-1, endothelial leukocyte adhesion molecule-1, inducible nitric oxide synthase, prostaglandin E2, and prostaglandin EP3 [69–72]. These mediators further induce a cascade of inflammatory events that have deleterious effects on islet graft survival.

In addition to several proinflammatory cytokines, islets also are known to express chemokines after isolation and during culture, of which CXCL10 (IP-10), CXCL8 (IL-8), and CCL2 (MCP-1) are the major ones [28, 43]. IP-10 is widely recognized as a chemokine that attracts leukocytes to the site of infection or inflammation. On the other hand, IL-8 and MCP-1 are known to recruit neutrophils and macrophages, respectively. The role of chemokines receptors and ligands in various transplant rejections has been reviewed elsewhere [73]. In the context of islet transplantation, it has been shown that allograft function is prolonged under anti-CXCL10 antibody therapy and this was due to the lack of infiltration of leukocytes to the graft site [74]. Using CCL2^−/−^ knockout mice, it was demonstrated that reducing CCL2 expression in recipients is more important than knocking out CCL2 in donors. Knocking out CCL2 in the recipient also resulted in abrogation of other chemokines (CXCL1, CCL4, CCL6, and CXCL9) that were upregulated in the liver after transplantation [75]. A recent approach using reparixin, an inhibitor of CXCR1/2, has shown improved islet transplant outcome in allogeneic mouse models. Furthermore, a pilot study on human islet allotransplant patients also showed promising results. Comparison of reparixin-treated group with nontreated controls showed reduced insulin requirement, less HbA1c levels after transplantation, and also improved fasting C-peptide levels. The proposed mechanism is the lack of recruitment of PMN (polymorphonuclear) leukocytes and NKT cells to the liver [76]. Based on these reports, it is conceivable that reducing secretion of proinflammatory cytokines and chemokines during the initial stages of transplantation could improve islet transplant outcomes.

Several reports have shown that allogeneic islet transplant recipients develop antibodies to both human leukocyte antigen (HLA) classes I and II antigens of donor origin [77–79]. While it is evident that islets constitutively express HLA class I antigens, the expression of HLA class II on isolated human islets has not been clearly demonstrated. Upon stimulation with inflammatory cytokines of TNF-*α* and IFN-*γ*, islet-enriched pancreatic cultures and MIN6 cell lines have been shown to express HLA class II antigens [80, 81]. Recently, our group has demonstrated that under inflammatory conditions, there is induction of HLA class II expression on the islet cell surface leading to the development of antidonor class II antibodies in the recipient [82]. Islets isolated from deceased donor pancreases were used to demonstrate induction of HLA class II molecules through real-time polymerase chain reaction analysis, immunofluorescent staining, and flow cytometry. One of the transplant recipients developed antibodies to a mismatched donor HLA class II allele (DR7). Posttransplant serum from this patient showed significant binding to cytokine-stimulated islet cells from DR7-positive donors only. These results clearly demonstrate that human islets express HLA class II under inflammatory conditions. This could lead to an anticlass II response, which in turn may play a critical role in rejection of islet allografts.

## 7. Posttransplant Inflammation

### 7.1. Hypoxia

Like other transplantable organs, islets are prone to ischemic reperfusion injury. When isolated islets are infused into the blood circulation, several cytotoxic products are produced due to oxygen metabolism, which is catalyzed by xanthine oxidase [83]. Activation of this enzyme results in the formation of superoxide and hydrogen peroxide, which can interact with free iron and copper radicals to produce cytotoxic reactive oxygen species (ROS) [84]. ROS can induce cell death by damaging and degrading proteins, lipids, and nucleic acids and activating NF-*κ*B inflammatory pathways [85]. Beta cells of the islets are more vulnerable to attack by ROS because of their low expression of antioxidants, including glutathione peroxidase, catalase, and superoxide dismutase [86–88]. Indeed, increasing the expression of cellular antioxidants and treatment with exogenous antioxidants have both been shown to protect islets in a diabetes models [89, 90].

Following intraportal infusion, there is a low oxygen supply at the graft site in the liver, creating a hypoxic environment for the islets [91, 92]. Islet cells have countermechanisms to restore the oxygen supply [22]. Eventually, islet grafts begin to vascularize, but the amount of oxygen supply even after hepatic vascularization is significantly lower than in the pancreas (5 mm Hg as opposed to 40 mm Hg) [93]. Although liver is currently the preferential site for islet transplantation, it is still not considered the optimal host site because of the lack of oxygen; therefore other sites that might improve the oxygen availability to the islets are still under consideration. Cantarelli et al., for example, have provided evidence that bone marrow may be a more suitable graft site for islet transplantation because of the increased vasculature and abundant supply of oxygen [94]. Furthermore, pilot studies on clinical autologous islet transplants have been performed in bone marrow which outperformed islet grafts in the liver [95].

### 7.2. Innate Immunity

Pancreatic islet inflammation after transplantation is induced by innate immune cells such as neutrophils, islet resident macrophages, and Kupffer cells. Kupffer cells can induce damage to other cells by releasing free radicals and secreting proinflammatory cytokines [96]. The amount of proinflammatory cytokines IL-1*β* and TNF-*α* secreted by Kupffer cells is increased after islet transplantation [97]. This inflammatory cytokine elevation is blocked in the case of a macrophage-depleted recipient. Several factors have been postulated to explain the activation of Kupffer cells in the liver after islet transplantation. One report suggested that the collagenase used for islet isolation contains endotoxin, which could activate production of proinflammatory cytokines by islet-resident macrophages and in turn activate Kupffer cells [98]. The presence of acinar tissue in the islet transplant preparation has also been investigated as the cause for activation of Kupffer cells. It has been demonstrated that the amount of exocrine contamination is directly correlated to posttransplant necrosis and lower viability of pancreatic islet cells. It has been postulated that when these acinar cells die, they release enzymes that trigger an inflammatory response leading to islet cell death [99]. Another hypothesis is that the professional APCs residing inside the islets are responsible for inducing the inflammatory response. A recent study on the intraislet immune cell population showed that 67% of the immune cells in the islets are APCs, and 50% of these APCs are B cells [100]. Use of anti-inflammatory agents that block the production and secretion of these proinflammatory cytokines and chemokine would be a useful strategy to protect islets from innate immune cell-mediated damage. Recently we have shown that use of Withaferin A, which is an NF*κ*B inhibitor, protected islet viability, reduced secretion of proinflammatory chemokines and cytokines, and reduced infiltration of neutrophils in a tube model [101].

## 8. Inflammatory Response from Islets

Islets can also produce inflammatory mediators in response to metabolic, inflammatory, physical, and chemical stress. For example, IL-1*β* induces production of cytokines, chemokines, and iNOS in pancreatic beta cells [102–104]. In the context of islet transplantation, multiple islet stress events may contribute to cascading cytokine-induced cytokine production in islets. Thus, cytokines produced and released by beta cells throughout the islet transplant process may exacerbate islet inflammation.

MAP kinases ERK1/2, p38, and JNK have roles in islet dysfunction and apoptosis in response to stress-specific signaling pathways [105, 106]. Calcineurin-dependent ERK1/2 activation has short-term stimulatory effects on insulin production and islet function in response to glucose and secretagogues; however, sustained activation due to metabolic and inflammatory stress may have deleterious effects [107–110]. Upon exposure to cytokines, JNK and p38 are activated by MAP kinase kinase (MKK) 4 and MKK3/6, respectively [106]. Additionally, exposure of islets to stress from the islet isolation process activates JNK and p38 via MKK7 signaling. One study showed that an increased p38/JNK activation relative to ERK1/2 activation after islet isolation correlates with decreased islet survival [111]. Hence, identifying methods to selectively block p38 and JNK pathways, while preserving acute ERK1/2 signaling, may enhance islet graft function and survival in islet transplantation [112–116].

Acute exposure of islets to low dose of IL-1*β* has been shown to stimulate proliferation and enhance beta-cell function, whereas exposure to high dose of IL-1*β* impairs secretion and induces apoptosis [117–119]. Induction of TNF-*α* expression in beta cells by IL-1*β* requires activation of calcineurin and downstream transcription factor nuclear factor of activated T cells (NFAT) [102]. Extended exposure of beta cells to IL-1*β* results in sustained MAP kinase signaling, which activates nuclear factor-kappa B (NF-*κ*B-)dependent genes that are proapoptotic [102, 120, 121]. Although a number of reports suggest that NF-*κ*B is responsible for apoptosis of transplanted islet cells [122], other reports have presented conflicting evidence suggesting that NF-*κ*B prevents islet cell death [123]. This is also likely attributed to the strength and duration of exposure as well as the type of stimulus, as both NFAT and NF-*κ*B have overlapping roles in cytokine gene expression and beta-cell proliferation and function [102, 123–126]. Thus, minimizing sustained activation of MAP kinase and NFAT/NF-*κ*B signaling during the transplantation process is likely key to reducing islet inflammation and improving islet transplant outcomes.

## 9. Anti-Inflammatory Strategies to Improve Islet Transplantation

Anti-inflammatory agents have been incorporated in immunosuppressive regimens in recent clinical allogeneic islet transplant protocols [127, 128]. A recent review has summarized progress related to this approach [14]. In the era of 1999 to 2002, TNF-*α* inhibitors were utilized in a small proportion (11.8%) of islet transplants, while in the 2007 to 2010 period, they were used in 33.8% of transplants [129]. The first report of islet transplantation involving an anti-inflammatory agent came from the University of Minnesota, where etanercept was incorporated in peritransplant immunosuppression therapy [130]. All the islet recipients achieved insulin independence after a single islet infusion. The University of Miami group implemented an immunosuppression protocol containing infliximab, which resulted in a high success rate of insulin independence (87.5%) [131]. Recently, a meta-analysis showed significant improvement in the insulin independence rate when a TNF-*α* inhibitor was coupled with a T-cell depletion regimen, compared with T-cell depletion only [132]. The success rate with T-cell depletion immunosuppression plus a TNF-*α* inhibitor is now likely comparable to that of whole-organ pancreas transplantation alone, offering approximately 50% success at 5 years after transplant [129, 132, 133].

No significant difference in the success rate, however, was seen between a T-cell-depleting islet protocol with and without infliximab in a clinical study [131]. In an immunodeficient rodent model transplanted with a marginal mass of human islets, no significant benefits in islet engraftment were observed when only etanercept was given, whereas significant improvement in diabetes reversal was demonstrated when both etanercept and anakinra, an IL-1*β* receptor antagonist, were administered [134]. Thus, administration of a TNF-*α* inhibitor alone might be insufficient to fully control peritransplant inflammation. Interestingly, our group has implemented combined therapy of etanercept and anakinra in clinical allogenic islet transplantation and has shown that all patients achieved insulin independence after a single infusion, although the number of islet recipients was limited [135]. A large, well-designed clinical study is required to evaluate the impact of anti-inflammatory agents in islet engraftment. The use of anti-inflammatory agents in clinical allogeneic islet cell transplantation is summarized in Table 1.

In our recent study, we have shown that a marginal dose of withaferin A, an anti-inflammatory compound and a potent NF-*κ*B inhibitor, can significantly improve graft survival in a syngeneic mouse islet transplant model. The islet damage due to proinflammatory cytokines and apoptosis was abolished [116]. The use of an NF-*κ*B inhibitor to reduce islet inflammation and protect islets from apoptosis is helpful in improving graft function and survival. Since NF-*κ*B is a critical transcription factor that can induce the expression of various proinflammatory cytokines, its activation needs to be prevented during transplantation until the graft revascularization is complete.

Identification of islet-specific biomarker(s) is necessary to evaluate islet damage by inflammation. Recently, high-mobility group box 1 (HMGB1) protein was shown to be released specifically from islet grafts immediately after transplant in a syngeneic rodent model [136]. The HMGB1 release was also observed following cytokine stimulation, and anti-HMGB1 treatment was able to restore glycemic control in diabetic mice with a marginal mass of syngeneic islet grafts. Furthermore, similar findings were seen in a study using human islets, which released a higher amount of HMGB1 under hypoxic conditions* in vitro*, corresponding to the degree of apoptosis [137]. In human autologous islet transplantation for patients with CP, significant elevation of serum HMGB1 levels was also observed during islet infusion, although no elevation of HMGB1 levels was found in patients who underwent total pancreatectomy alone [138]. Interestingly, there was a significant difference in changes in HMGB1 levels during the peritransplant period between patients achieving insulin independence and those who continued to require insulin therapy after transplant. These findings suggest that serum HMGB1 levels can be a useful clinical biomarker to evaluate islet damage.

Several anti-inflammatory compounds are currently being investigated and more emerging to improve the outcome of islet transplantation. A very exhaustive review on the strategies and approaches to improve clinical islet transplantation has been mentioned elsewhere [14]. Pileggi et al. have shown that expression of heme oxygenase-1 (HO-1) in islet cells protected the islets from apoptotic death. To induce HO-1 in islets, the cells were cultured in ferriprotoporphyrin IX chloride and cobaltic protoporphyrin IX chloride before transplantation [139]. The HO-1 gene plays an important role in iron homeostasis but also behaves as a potent anti-inflammatory and antiapoptotic gene [140, 141]. A similar study has shown the antiapoptotic effect when islets are treated with carbon monoxide. This treatment causes islets to produce cytoprotective cyclic guanosine monophosphate and its downstream kinases [142]. Another strategy that has shown promise is the expression of the A20 gene in islets by* ex vivo* gene transfer, which protects these islets from cytokine-induced damage. A20 is an inhibitor of NF-*κ*B, which is a transcriptional regulator of several proinflammatory cytokines [143]. A different study involving the use of diannexin, which is a homodimer of annexin 5, has demonstrated a significant decrease in *β*-cell apoptosis and improved graft function after* in vivo* transplantation [144]. The use of JNK inhibitor SP600125 in combination with nicotinamide and simvastatin protected porcine islets from peritransplant inflammation and apoptosis. Intraductal administration of JNK inhibitor protected islets by inhibiting the production of proinflammatory cytokines IL-1*β*, TNF-*α*, IFN-*γ*, IL-6, IL-8, and MCP-1* in vivo *[145].

## 10. Future Direction

Inflammation during islet transplantation is multifaceted and the underlying mechanisms are quite diverse. Several factors could play critical roles in the induction or exacerbation of inflammation. Therefore, it is challenging to identify one particular approach or strategy to eliminate all inflammatory events in islets. A combinatorial approach based on the current understanding of inflammatory mechanisms will likely be required to improve islet transplant outcomes. Overall, isolation of islets from pancreas with less ischemic time is an important aspect. Furthermore, culturing islets in conditions that are suitable for islet survival and use of antioxidants and anti-inflammatory compounds including potent inhibitors of NF*κ*B, which are known to regulate several proinflammatory genes, is important. Since a significant mass of islets is immediately lost to IBMIR, major focus should be to reduce this response. Although the use of anticoagulants or complement inhibitors shows benefits in* in vitro* and* in vivo* settings, they fail to perform effectively in the clinical settings. Moreover, in allogenic transplants, the inflammation is severe and therefore a combined anti-inflammatory regimen to reduce ensuing immune response would be required. For instance, LMWDS has shown potential benefits to prevent clotting. This could be combined with anti-inflammatory compounds such as anakinra, etanercept, and with complement inhibitors to form a complete regimen to block IBMIR. Some of the recent clinical studies, such as use of reparixin to block CXCR1/2, have shown promising outcomes. Several inflammatory mechanisms that cause dysfunction of islets that have been discussed here should be addressed at the clinical level. Future studies should focus on these mechanisms to develop strategies for successful islet transplantation.

## 11. Conclusion

The inflammatory response has been shown to be a major cause of posttransplant islet graft failure in both an experimental animal model and in clinical studies. Inflammation can be triggered by multiple factors: brain death in the pancreas donor, ischemia during pancreas preservation, physiologic and enzymatic stress during islet isolation, islet infusion, and recipient's innate immunity. TNF-*α* inhibition has been demonstrated as a potent anti-inflammatory strategy in clinical allogeneic islet transplantation, although other agents, including NF-*κ*B inhibitors, have also been studied for the prevention of an inflammatory reaction in experimental models. More precise and deeper understanding of the inflammatory response to transplanted islets is warranted. Control of inflammation could improve the efficacy of islet cell transplantation, which in turn could enhance the long-term function of transplanted islets.

## Figures and Tables

**Table 1:** Anti-inflammatory agents in clinical allogeneic islet cell transplantation.

Publication year	Pt number	Anti-inflammatory agents	Dose of anti-inflammatory agents	Induction therapy	Maintenance therapy	Major outcomes	References
2005	8	Etanercept	50 mg i.v. pretransplant and 25 mg s.c. on days 3, 7, and 10	rATG Daclizumab	Sirolimus Tacrolimus MMF	100% II after single infusion	[130]

2005	8	Infliximab	5 mg/kg i.v. pretransplant	Daclizumab	Sirolimus Tacrolimus	7/8 recipients achieved II	[131]

2008	7	Infliximab or Etanercept	5 mg/kg i.v. pretransplant or 50 mg i.v. pretransplant and 25 mg s.c. twice weekly for 2 weeks	Daclizumab	Sirolimus Tacrolimus (Low-dose steroids for 3 pts)	6/7 recipients achieved II	[146]

2008	3	Etanercept	50 mg i.v. pretransplant and 25 mg s.c. twice weekly for 2 weeks	Alemtuzumab	Sirolimus Tacrolimus MMF	2/3 recipients achieved II	[147]

2008	6	Etanercept	50 mg i.v. pretransplant and 24 mg s.c. on days 3, 7, and 10	rATG (for first transplant) Daclizumab (for 2nd transplant)	Cyclosporine Everolimus	5/6 recipients achieved II	[148]

2008	6	Etanercept	50 mg i.v. pretransplant and 24 mg s.c. on days 3, 7 and 10	Daclizumab	Sirolimus Tacrolimus	6/6 recipients achieved II	[149]

2011	3	Etanercept and anakinra	50 mg i.v. pretransplant and 25 mg s.c. on days 3, 6, and 10 and 100 mg i.v. pretransplant and 100 mg s.c. for 7 days after transplant	rATG	Tacrolimus MMF	3/3 recipients achieved II	[135]

2012*	22	Anti-TNF-*α*	NA	T-cell depletion protocol	NA	50% of recipients kept II for 5 years	[132]

i.v. indicates intravenous injection; s.c., subcutaneous injection; rATG, rabbit antithymocyte globulin; MMF, mycophenolate mofetil; II, insulin independence; TNF, tumor necrosis factor.

*Based on collaborative islet transplant registry, collecting data from multiple centers.
